# The relationship between involvement in group extreme conditioning program training and PERMA: the mediating role of social capital

**DOI:** 10.3389/fpsyg.2026.1796533

**Published:** 2026-04-16

**Authors:** Apostolia Ntovoli, Georgia Stavropoulou, Garyfallos Anagnostou, Elena Papacosta, Sousana Papadopoulou, Constantinos Giaginis, Kostas Alexandris

**Affiliations:** 1 School of Health Sciences, Department of Life and Health Sciences, Frederick University, Limassol, Cyprus; 2 Laboratory of Management of Sports, Recreation, and Tourism, Department of Physical Education and Sport Sciences, School of Physical Education and Sport Science, Aristotle University of Thessaloniki, Thessaloniki, Greece; 3 Department of Nutritional Science and Dietetics of International Hellenic University (IHU), Thermi, Greece; 4 Department of Food Science and Nutrition, School of Environment, University of the Aegean, Mytilene, Greece

**Keywords:** extreme conditioning program training, involvement, PERMA, social capita, subjective well being

## Abstract

**Introduction:**

Group exercise in the form of an extreme conditioning program training can have positive psychological outcomes for participants. This study aims to investigate whether involvement in this kind of program affects the development of subjective well being, as measured by the PERMA profiler, considering social capital as a mediator in the relationship between involvement and subjective well being.

**Methods:**

The data were collected through an online survey of adults who participated in an extreme conditioning program training in Greece, with a sample of 590 individuals. Involvement was measured using a three-dimensional model: centrality, attraction, and self-expression. Social capital was measured based on the three domains: network, trust, and reciprocity. Finally, subjective well being was measured with the PERMA profiler, including the dimensions of positive emotions, engagement, positive relationships, meaning, and accomplishment. Aiming to explore the direct and indirect effects of attraction, centrality, and self-expression on positive emotions, engagement, relationships, meaning, and accomplishment, via social capital, a mediation analysis was conducted. The mediation model was assessed through path coefficients, and indirect effects were examined to ascertain the degree to which social capital mediated the relationship between attraction, centrality, self-expression, and well being dimensions.

**Results:**

The results indicated moderate to strong statistical relationships between involvement and social capital, as well as between social capital and well being. Furthermore, social capital fully mediated the relationship between centrality and well being and partially mediated the relationship between attraction and well being. These results support the value of group exercise in the form of an extreme conditioning program.

**Conclusion:**

These results support the value of group exercise in the form of extreme conditioning program training in developing social capital and positively influencing participants' subjective well being.

## 1 Introduction

Group exercise in the form of extreme conditioning program training has evolved into a very popular training practice and a global fitness paradigm due to its physical and psychological benefits (Rocha et al., 2025; Tibana and de Sousa, 2018). As a result, it has attracted significant academic interest (Ferdinando, 2025).

As a strength and conditioning model, group fitness programs based on the principles of extreme conditioning training were developed around the following domains: cardiovascular/respiratory endurance, stamina, strength, flexibility, power, speed, coordination, agility, balance, and accuracy (Claudino et al., 2018). They are characterized by rapid, repetitive, high-intensity efforts performed with minimal or absent recovery intervals (Sprey et al., 2016). Despite the widespread spread of extreme conditioning program training and the physical benefits that have been reported, there has also been some criticism, related to reported injury incidences, particularly among novices performing technically demanding lifts such as snatches or muscle-ups without sufficient mastery (Hak et al., 2013; Weisenthal et al., 2014). Moreover, the intensity of such programs raised concerns regarding overtraining or overuse, with documented injury rates in several epidemiological studies (Feito et al., 2019, 2020; Rios et al., 2024) and systematic reviews (Rodríguez et al., 2022).

While research on the physiological benefits and risks associated with extreme conditioning program training is extensive (Claudino et al., 2018; Rios et al., 2024), there is still limited research on its psychosocial outcomes (Amato et al., 2025; Whiteman-Sandland et al., 2016). We argue that the successful business model that led to the worldwide expansion of such programs might also be due to the psychosocial benefits associated with it, such as the development of social capital, individual well being and collectively societal well being (United Nations, 2024). Extreme conditioning program training in groups can promote friendship, mutual understanding, and a more peaceful society. It can bring together people from diverse cultural backgrounds and allow them to interact with each other through exercise participation. Both these can be included under the umbrella of “social sustainability”.

It has been reported that more than just a training system, such programs are consolidated into a distinct cultural formation, characterized by a pronounced community-driven ethos and the pursuit of individual well being and health-oriented lifestyles (Whiteman-Sandland et al., 2016). Furthermore, their conceptual simplicity and group training format have facilitated the generation of substantial social interaction and communities, expressed through networks of practitioners, coaches, and affiliated clubs, which not only promote participation but also contribute to the development of a committed participant base (Amato et al., 2025; Gao et al., 2025; Prochnow et al., 2022).

This paper, for the first time, examines whether involvement in group extreme conditioning program training can develop social capital and if social capital mediates the relationship between involvement and perceived well being. On measuring perceived well being, we used the PERMA profiler (Seligman, 2011), which is a holistic measure, incorporating both the hedonic and eudemonic elements of well being (Huta and Waterman, 2014). While the relationship between exercise participation and individual well being is well established (Biddle and Asare, 2011; Chekroud et al., 2018), there have been very limited attempts so far to test the influence of social capital on perceived well being (Lin et al., 2024; Smith et al., 2023), in an integrated model, measuring at the same time the level of participants' involvement. We propose that the role of social capital is particularly important in the context of group exercise, given its community-delivery focus and the promotion of club/participant networks (Brown and Jones, 2022). This paper contributes to literature in two ways. First, it tests, for the first time, the interactions among involvement, social capital, and perceived well being, all measured using multidimensional approaches. Second, from a contextual aspect, it measures psychosocial outcomes of involvement with extreme conditioning program training (social capital and perceived well being), aiming to explain their widespread expansion and the development of such a committed participant network (Chen et al., 2023; Wang et al., 2024), and provide empirical evidence for the value of group exercise in the form of extreme conditioning program training on addressing the sustainable development goals (e.g., SDG 3 and SDG 16).

This study is the first to examine whether involvement in a group extreme conditioning program training fosters social capital, and whether social capital mediates the relationship between involvement and perceived well being. Perceived well being was measured using the PERMA profiler (Seligman, 2011), a holistic instrument that captures both hedonic and eudaimonic dimensions of well being (Huta and Waterman, 2014). Although the positive association between exercise participation and individual well being is well established (Biddle and Asare, 2011; Chekroud et al., 2018), limited research has explored the influence of social capital on perceived well being within an integrated model that simultaneously accounts for participants' level of involvement (Lin et al., 2024; Smith et al., 2023). We argue that social capital plays a particularly salient role in group exercise contexts due to their community-based delivery and the cultivation of club- and participant-level networks (Brown and Jones, 2022).

This paper contributes to the literature in two ways. First, it empirically examines—for the first time—the interplay among involvement, social capital, and perceived well being within the context of group extreme conditioning program training. By operationalizing each construct through multidimensional measures, the study advances existing research that has typically treated these variables in a unidimensional manner. This integrated approach allows for a more nuanced understanding of how involvement with an activity translates into psychosocial outcomes, and specifically how social capital functions as a mediating mechanism linking participation to well being. Second, from a contextual standpoint, the study evaluates the psychosocial outcomes associated with participation in extreme conditioning program training, focusing on the development of social capital and the enhancement of perceived well being. These insights help explain the rapid global expansion of such programs and the emergence of highly committed exercise communities characterized by strong interpersonal ties, shared norms, and collective identity (Chen et al., 2023; Wang et al., 2024).

## 2 Theoretical background

### 2.1 Group extreme conditioning program training and psychosocial outcomes

While research on the physiological outcomes of extreme conditioning program training is extensive (Claudino et al., 2018), there is still limited research on the psychosocial outcomes. As noted, one of the key aspects of such training is its community-oriented structure, which has been identified as a key mechanism underpinning enhanced motivation and increased commitment levels (Whiteman-Sandland et al., 2016). It is well documented today that group-based training formats reinforce social cohesion and promote solidarity through collective effort and shared trials (Feito et al., 2019; Prochnow et al., 2022), addressing also the Sustainable Development Goal (SDG) 16 “promote peaceful and inclusive societies”. This is particularly manifested in competitive arenas such as extreme conditioning program training events that take place around the world (Prochnow et al., 2022).

This communal dynamic is further amplified by digital platforms, where participants circulate their performances and personal achievements, thereby constructing transnational circuits of accountability and recognition. Group-based sessions, partner WODs such as Cindy performed in pairs, and community events, including charity competitions, also generate strong social ties (Prochnow et al., 2022) and promote peaceful and inclusive societies as defined by SDG 16. Such practices promote social interaction, networking, and build personal identities (Dawson, 2025; Ntovoli et al., 2025). Whiteman-Sandland et al. (2016), for example, reported that 78% of long-term participants identified social support as their primary motivator, often describing their local exercise community as a “second family.”

Scalability that reinforces psychological needs is another distinctive feature of these kinds of fitness programs. By allowing workouts to be adapted to individual capacities—for instance, replacing pull-ups with ring rows for novices or adding load to thrusters for advanced practitioners— such training fosters autonomy and progressive mastery (Browne et al., 2022). This flexibility strengthens perceptions of competence, as noted by Davies et al. (2016), who found that 65% of participants reported enhanced self-efficacy after 6 months of training, citing measurable progress in benchmark workouts. The public display of WOD performance, often through gym whiteboards, further transforms abstract goals into tangible achievements, leading to motivational reinforcement. These increased levels of motivation can be explained by the Self-Determination Theory (SDT; Deci and Ryan, 2008), which highlights autonomy, competence, and relatedness as essential drivers of motivation (Dominski et al., 2020). These dimensions are deeply embedded in extreme conditioning program training, enabling participants to feel empowered, capable, and socially connected, which collectively are an integral part of social well being, as defined by SDG3. It has been reported that this social dynamic and increased motivation levels mitigate monotony and significantly reduce dropout rates, with attrition in extreme conditioning program training gyms (~15%) markedly lower than in traditional, typical fitness centers (~50%; Claudino et al., 2018). It has, however, to be noted that the literature also underscores potential psychosocial risks, such as tendencies toward exercise dependence and body image concerns, with heightened prevalence among female participants (Coyne and Woodruff, 2020).

Finally, research has also shown that group extreme conditioning program training can positively influence mental health, which is an important element of individual well being. Training sessions can stimulate endorphin and dopamine release, neurotransmitters associated with motivation and positive effect (Basso and Suzuki, 2017). Such responses are linked with reductions in anxiety and depression. Wilke (2020) demonstrated that a single extreme conditioning program training session improved working memory and inhibitory control by 12–15%, outperforming moderate-intensity aerobic activity. Longitudinal investigations confirm sustained outcomes, showing that regular participants report approximately 30% lower stress and 25% higher life satisfaction compared to non-participants (Zhang et al., 2025). Furthermore, such training contributes positively to self-perception. Research by Coyne and Woodruff (2020) examined the program's role in shaping women's body image, self-esteem, and eating behaviors, while also identifying motivational drivers and environmental preferences linked to continued participation.

### 2.2 Social capital in sports

Social capital has been a popular topic for academics and policy makers during the last 20 years, since it is well documented today that it is linked with increased physical, social and psychological benefits for individuals, but also social connectivity, social and emotional skills, and community building, contributing finally to healthy societies (Forsell et al., 2020; Smart, 2000; Zhang et al., 2025), and it is included in several of the SDGs, such as SDG3, SDG5 and SDG16. There are several definitions of social capital in the literature. (Putnam 2000, p. 19) defined social capital as “social networks and the norms of reciprocity and trustworthiness that arise from them”, while Lin (2002) defined social capital as the “investment of resources with expected returns in the marketplace.” (Lin et al., 2001). These returns were named as “benefits” by Portes (2000), which was later substituted with the term “resources”. In this line, social capital is seen as the development of social networks that provide individuals with assets that can be used at a community and societal level (Bourdieu, 1986; Field, 2008; Ntovoli et al., 2025).

Putnam (1995) defined these assets as “the features of social organization such as networks, norms, and social trust that can facilitate coordination and cooperation for mutual benefit” (p. 66)

and conceptualized them on three core elements: network, trust, and reciprocity. Network is the core element of social capital, because it describes the structural relationships developed among the members of a social group (Zhou and Kaplanidou, 2018), which are the exercise groups and fitness clubs of the extreme conditioning program training. Trust is a prerequisite of the development of social networks, as it is impossible to have social interaction and exchange without it (Lewandowski, 2018; Portes, 2000, 2024). It has been proposed that trust can be developed by people who know each other and between strangers (Scrivens and Smith, 2013). In the fitness context, members of training groups obviously know each other as they spend training time together and share common goals. Reciprocity refers to a social norm that involves members' willingness to exchange benefits and help to achieve common goals (Prayitno et al., 2024). This is particularly applicable in the case of extreme conditioning program training, as members help each other to achieve personal and group training goals. This help can take the form of support, which is an important motivating factor (Ntovoli et al., 2025), but also the exchange of knowledge and experience. It is well documented today that sport participation contributes to the development of social capital because sport engagement can help the development of social networks, social connectiveness, and a sense of belonging (Kim et al., 2021; Kumar et al., 2018; Zhou and Kaplanidou, 2018; Zhou et al., 2021). It should be noted that social capital can be both an outcome and an antecedent of sport participation. It is still not clear today whether sport participation promotes social capital or if individuals with a higher level of social capital are more likely to take part in sports (Kim et al., 2021).

### 2.3 Involvement, subjective well being and social capital

Prior research has shown that higher involvement in physical activity is associated with stronger psychological commitment, greater satisfaction, and more positive affective outcomes (e.g., Biddle and Asare, 2011; Chekroud et al., 2018).

It must be noted that involvement in the current study was treated as an attitudinal construct, describing an individual's level of interest and devotion to a specific sport activity (Funk et al., 2022). The three-dimensional model proposed by Kyle et al. (2003) was used, including Attraction, Centrality, and Self-expression. Attraction is expressed when a sport activity is perceived as fun, interesting, and enjoyable. Centrality refers to the degree to which a sport activity has an important role in an individual's everyday life. Finally, self-expression refers to the degree to which an individual identifies with a specific sport activity, which is used to express her/his personal identity. Research on sport involvement in the last 20 years has been extensive (Funk et al., 2022). It is not the objective of this paper to review the sport involvement literature, since it was used only as an independent variable in this study. Within the context of group extreme conditioning program training, these dimensions are particularly salient, as participants often develop strong emotional bonds with the activity, integrate it into their daily routines, and use it as a means of expressing personal values and identity. Given the multidimensional nature of involvement and the holistic conceptualization of well being adopted in this study, it is reasonable to expect that greater involvement will be associated with higher levels of perceived well being across its various components. Based on this discussion, the first hypothesis was set:

*H1: Involvement—operationalized through attraction, centrality, and self-expression—in group extreme conditioning program training positively influences the development of perceived well being, including positive emotions, engagement, positive relationships, meaning, and accomplishment*.

As previously noted, social capital refers to the resources embedded within social networks, including trust, reciprocity, shared norms, and social connectedness. Group exercise settings are particularly conducive to the development of social capital because they foster repeated interactions, shared experiences, and collective identity formation (Brown and Jones, 2022). Individuals with higher involvement are more likely to participate consistently, interact with peers, and invest in the social environment of the group, thereby strengthening their social ties and sense of belonging. Prior studies have suggested that involvement in leisure and sport activities can enhance bonding and bridging social capital (e.g., Lin et al., 2024; Smith et al., 2023), yet this relationship has not been empirically tested in the context of extreme conditioning programs. Accordingly, we propose the following second hypothesis.

*H2: Involvement in a group extreme conditioning program training positively influences the development of social capital*.

Research has shown that social capital can have positive outcomes both at the individual and societal levels. In a detailed discussion of the different types of social capital as outcomes of sport participation, Widdop et al. (2016) categorized them into three categories: individual level, community level, and national level. At an individual level, sport participation helps individuals to develop social relationships, satisfy their needs and personal goals, contributing to individual well being. At the community level, sport participation provides opportunities for building social networks, bringing participants together, and increasing community engagement. Finally, at a national level, sport participation can help individuals to exchange social norms through sport participation, promote fairness, and interact with different cultures, especially in the cases of international sport events. All these three levels are in line with SDGs 3, 5, and 16.

While the relationship between social capital and societal benefits is well documented today, there is still limited research on the individual psychological benefits of social capital (Zhang et al., 2025). In a systematic review of the published studies, Zhang et al. (2025) reported that some studies have found positive relationships between social capital and hedonic well being, while there have been studies that reported no relationships. Downward et al. (2018) reported a significant association between social capital and hedonic well being at the individual level. However, Kumar et al. (2018) contested this relationship, finding that social capital and hedonic well being had only an indirect relationship, which is mediated by health. These contradictory findings might also be due to the complexity of defining and measuring social capital and hedonic well being, especially within the context of sports and physical activities (Zhang et al., 2025).

In the current study, we tested the relationship between social and subjective well being measured with the holistic approach of the PERMA profiler. Subjective well being refers to “the degree to which people have positive appraisals and feelings about their lives, considered as a whole” (Fuhrer, 2000, p. 483). High levels of well being are associated with healthy behaviors, including social integration, increased leisure time, and involvement in sports and tourism (Lyubomirsky et al., 2005; Ntovoli et al., 2025; Theodorou et al., 2024). It has been proposed that well being has both an eudemonic (cognitive) and a hedonic (affective) element (Huta and Waterman, 2014). Seligman (2011) proposed a holistic approach to defining and measuring perceived well being: the PERMA profiler, which includes both the hedonic and eudemonic elements. Five pillars were proposed to define perceived well being: positive emotions, engagement, positive relationships, meaning, and accomplishment. Positive emotions are linked with happiness and enjoyment in an individual's life (Butler and Kern, 2016), which can be promoted with fun, active sport and exercise participation (Filo and Coghlan, 2016; Funk et al., 2022). Engagement refers to individuals' psychological immersion and absorption in an activity, reflecting the extent to which they experience focus, involvement, and flow during participation (Alexandris et al., 2017; Zhou and Kaplanidou, 2018). In contrast, positive relationships capture the interpersonal dimension of well being, encompassing social integration, belonging, and acceptance within one's social networks (Brajša-Žganec et al., 2011; Coleman and Iso-Ahola, 1993; Ntovoli et al., 2026). These relational aspects are particularly salient in sport and exercise contexts, which provide structured and informal opportunities for socialization, community building, and the development of supportive social ties (e.g., through social and community clubs; Coghlan and Filo, 2013; Filo et al., 2008; Funk et al., 2011). Meaning is linked with an individual's perceptions of having a meaningful life that contributes to community and societal well being. These perceptions are particularly manifested in the cases of volunteerism, charities, and social work (Doyle et al., 2016). Finally, accomplishment is linked with individual success and achievement of personal goals in several settings in an individual's life, including achieving goals in sport and exercise participation. The PERMA profiler is a valid and reliable framework, having been tested in various contexts such as education (Butler and Kern, 2016), sport participation (Lyubomirsky et al., 2005; Ntovoli et al., 2025), sport fans (Doyle et al., 2016), and sport events (Filo et al., 2022).

Summarizing, while previous research has explored the relationship between social capital and hedonic aspects of well being in exercise settings, as noted, —typically focusing on affective outcomes such as enjoyment, positive emotions, or momentary affective states (e.g., Lin et al., 2024; Smith et al., 2023)—it has not examined the broader and more comprehensive link between social capital and subjective well being in a more holistic perspective, which is the central focus of this study. This gap warrants attention because subjective well being encompasses both hedonic and eudaimonic components, reflecting individuals' overall evaluations of life satisfaction, meaning, and psychological functioning (PERMA; Huta and Waterman, 2014; Seligman, 2011). By addressing this gap, the present study advances literature beyond affective indicators and provides a more holistic account of the psychosocial benefits associated with participation in group-based extreme conditioning programs. This leads to our third hypothesis:

*H3: Social capital positively influences the development of perceived well being (positive emotions, engagement, positive relationships, meaning, and accomplishment)*.

As individuals become more involved in group extreme conditioning program training—through attraction, centrality, and self-expression—they tend to participate more consistently, interact more frequently with peers, and invest more deeply in the social environment of the group. These patterns of engagement create positive conditions for the development of social capital, including trust, reciprocity, shared norms, and a sense of belonging (Lin et al., 2024; Brown and Jones, 2022; Smith et al., 2023). At the same time, social capital has been shown to enhance multiple dimensions of subjective well being by providing emotional support, fostering meaningful social connections, and reinforcing individuals' sense of purpose and accomplishment (Brajša-Žganec et al., 2011; Coleman and Iso-Ahola, 1993; Ntovoli et al., 2026). Prior research suggests that social capital can serve as a psychosocial resource that strengthens both hedonic and eudaimonic well being outcomes (Coghlan and Filo, 2013; Filo et al., 2008; Funk et al., 2011), yet this mechanism has not been empirically tested within the context of group extreme conditioning program training. Thus, social capital represents a possible pathway through which involvement translates into higher levels of perceived well being. Building on this theoretical logic, we propose the following hypothesis.

*H4: Social capital mediates the relationship between involvement—operationalized through attraction, centrality, and self-expression—and perceived well being, encompassing positive emotions, engagement, positive relationships, meaning, and accomplishment*.

**Figure 1:**
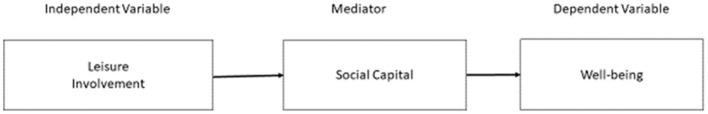
The theoretical model.

A graphical presentation of the theoretical model is presented in Figure 1.

## 3 Materials and research method

### 3.1 Procedure

The study followed a cross-sectional design. The data were collected with an online quantitative survey, including participants in a group extreme conditioning program training in Greece. An e-questionnaire was developed and distributed through social media (Facebook, LinkedIn, and blogs) to adults (>18 years old) who were participants in an extreme conditioning program. The data collection took place in the Autumn of 2025. Informed consent was obtained from all participants before completing the questionnaire. Five hundred and ninety questionnaires were collected. Since the questionnaire was administered online, all items were set as mandatory, resulting in no missing cases in the dataset. To further ensure sample validity, response patterns were checked by the researchers for straight-lining and other indicators of low-quality or non-differentiated answers. It must be acknowledged that the sampling method followed was a convenience and not a probability one. Subsequently, the results of the study are not representative of the study population and cannot be generalized with confidence. However, this sampling method was the most effective in achieving the sample size required to test the hypothesis of the study.

### 3.2 Participants

With a sample size of *N* = 590, the study was adequately powered to detect small indirect effects in mediation analyses (Fritz and MacKinnon, 2007). From the participants, 41.9% were men and 58.0% were women, with one participant not specifying their gender. The mean age of the group was 26.9 (SD = 7.1). Regarding marital status, most participants were single (67.2%), followed by married (26.3%) and divorced (6.5%). In terms of education, 35.3% had a bachelor's degree, 20.9% a master's degree, 31.1% completed secondary education, while smaller percentages had completed primary education (0.2%), a vocational institute (7.8%), or a technological educational institute (4.8%). Occupationally, 34.0% were students, 31.9% worked in the private sector, 17.7% were self-employed, 12.1% were public sector employees, with small percentages unemployed (3.1%), retired (1.0%), or homemakers (0.3%).

### 3.3 Instruments

Leisure involvement was measured with the Kyle et al. (2003) scale, adapted to the context of group extreme conditioning program training. This model includes three dimensions: Centrality (3 items), Self-expression (3 items), and Attraction (4 items). This scale has been used extensively in several different versions among studies in sport and leisure contexts (Alexandris et al., 2022; Funk et al., 2022; Ntovoli et al., 2025) and was shown to be valid and reliable. The model demonstrated very good fit, χ^2^ (13) = 52.45, *p* < 0.001, CFI = 0.99, TLI = 0.98, RMSEA = 0.07, 90% CI [0.05, 0.09], SRMR = 0.02. The internal consistency for Attraction was α = 0.97, for Self-expression α = 0.93, and for Attraction α = 93. Social capital was measured with nine items, which correspond to the three dimensions of social capital, as proposed by Putnam: network (3 items), trust (3 items), and reciprocity (3 items). The model demonstrated very good fit, χ^2^ (25) = 100.75, *p* < 0.001, CFI = 0.99, TLI = 0.98, RMSEA = 0.07, 90% CI [0.06, 0.09], SRMR = 0.03. The internal consistency for Network was α = 0.95, for Trust α = 0.88, and for Reciprocity α = 0.89. Special reference should be made to the scale of Zhou et al. (2021), who, based on these three dimensions, developed a nine-item sport social capital scale for participants in sport events. Subjective well being was measured with the PERMA profiler (Butler and Kern, 2016), including the five pillars: “positive emotions” (3 items), “engagement” (3 items), “positive relationships” (3 items), “meaning” (3 items), and “accomplishment” (3 items). This scale has been used in several studies in sport and exercise settings and has been shown to be reliable and valid (Filo et al., 2022; Karagiorgos et al., 2025; Ntovoli et al., 2025a,b; Oshimi and Kinoshita, 2025). In the current research the model demonstrated very good fit, χ^2^ (79) = 331.23, *p* < 0.001, CFI = 0.97, TLI = 0.96, RMSEA = 0.07, 90% CI [0.07, 0.08], SRMR = 0.03. The internal consistency for Positive Emotions was α = 0.93, for Engagement α = 0.81, for Positive Relationships α = 0.82, for Meaning α = 0.91, and for Accomplishment α = 92. The composite scores of these seven pillars were calculated to be used in our measurement and structural models. All the subscales were measured in seven-point Likert items ranging from 1—totally disagree to 7—totally agree.

### 3.4 Data analysis

The analysis was made with JASP software (via R). A set of regression models and mediation analysis was conducted to test the relationships between attraction, centrality, self-expression, well being, positive emotions, engagement, relationship, meaning, and accomplishment. First, descriptive statistics were utilized, followed by Pearson's correlation coefficients, which were used to examine the associations among the variables of interest and to elucidate the nature of their interrelationships. Aiming to explore the direct and indirect effects of attraction, centrality, and self-expression on positive emotions, engagement, relationships, meaning, and accomplishment, via social capital, a mediation analysis was conducted. The mediation model was assessed through path coefficients, and indirect effects were examined to ascertain the degree to which social capital mediated the relationship between attraction, centrality, self-expression, and well being dimensions. The model fit was evaluated using multiple indices, including chi-square (χ^2^), Comparative Fit Index (CFI), Tucker–Lewis Index (TLI), Root Mean Square Error of Approximation (RMSEA), and Standardized Root Mean Square Residual (SRMR). Furthermore, estimates of direct, indirect, and total effects were obtained, and their statistical significance was determined through bootstrapping procedures with 95% confidence intervals.

## 4 .Results

Descriptive statistics for all variables under examination are presented in Table 1.

**Table 1:** Descriptive statistics for attraction, centrality, self-expression, social capital, positive emotions, engagement, relationship, meaning, and accomplishment.

Variables	Min	Max	Mean	SD	Skewness	Kyrtosis
Attraction	1	7	5.18	1.79	−0.59	−0.92
Centrality	1	7	4.19	1.91	−0.02	−1.27
Self-expression	1	7	4.29	1.92	−0.08	−1.31
Well being	1	7	4.87	1.53	−0.59	−0.55
Positive emotions	1	7	5.07	1.48	−0.67	−0.34
Engagement	1	7	5.11	1.38	−0.71	−0.22
Relationship	1	7	5.01	1.46	−0.62	−0.40
Meaning	1	7	5.17	1.45	−0.81	−0.03
Accomplishment	1	7	5.22	1.40	−0.86	0.09

### 4.1 Correlations

Table 2 shows the correlations among Attraction, Centrality, Self-Expression, Social Capital, Positive Emotions, Engagement, Relationship, Meaning, and Accomplishment. All correlations were positive and statistically significant (all *p* < 0.001). Attraction showed moderate correlations with social and well being outcomes, including Social Capital (*r* = 0.58, *p* < 0.001), Positive Emotions (*r* = 0.52, *p* < 0.001), and Engagement (*r* = 0.51, *p* < 0.001). Centrality was strongly related to Self-Expression (*r* = 0.87, *p* < 0.001) and moderately associated with Social Capital (*r* = 0.58, *p* < 0.001) and Engagement (*r* = 0.49, *p* < 0.001), highlighting its role in both social and psychological constructs. Self-Expression was also positively associated with Social Capital (*r* = 0.56, *p* < 0.001) and Positive Emotions (*r* = 0.43, *p* < 0.001). Among well being variables, Positive Emotions, Engagement, Positive Relationships, Meaning, and Accomplishment were strongly intercorrelated (*r* ranging from 0.68 to 0.80, all *p* < 0.001), emphasizing the intertwined nature of these constructs. Table 2 provides the full correlation matrix for all variables. Table 2 presents the correlations found among all the study variables. To assess potential multicollinearity among predictors, collinearity diagnostics were conducted. The maximum Condition Index was 20.58, below the critical threshold of 30, and variance proportions did not indicate problematic multicollinearity. Therefore, all predictors were retained in the model.

**Table 2:** Correlations among attraction, centrality, self-expression, social capital, positive emotions, engagement, relationship, meaning, and accomplishment.

Variables	1.	2.	3.	4.	5.	6.	7.	8.	9.
1. Attraction	–								
2. Centrality	0.74^***^	–							
3. Self-expression	0.74^***^	0.87^***^	–						
4. Social capital	0.58^***^	0.58^***^	0.56^***^	–					
5. Positive emotions	0.52^***^	0.43^***^	0.43^***^	0.54^***^	–				
6. Engagement	0.51^***^	0.49^***^	0.47^***^	0.61^***^	0.77^***^	–			
7. Relationship	0.43^***^	0.38^***^	0.34^***^	0.53^***^	0.72^***^	0.69^***^	–		
8. Meaning	0.46^***^	0.39^***^	0.36^***^	0.56^***^	0.77^***^	0.72^***^	0.80^***^	–	
9. Accomplishment	0.47^***^	0.42^***^	0.39^***^	0.61^***^	0.72^***^	0.72^***^	0.68^***^	0.79^***^	–

^***^p < 0.001.

### 4.2 Social capital as mediator

**Figure 2:**
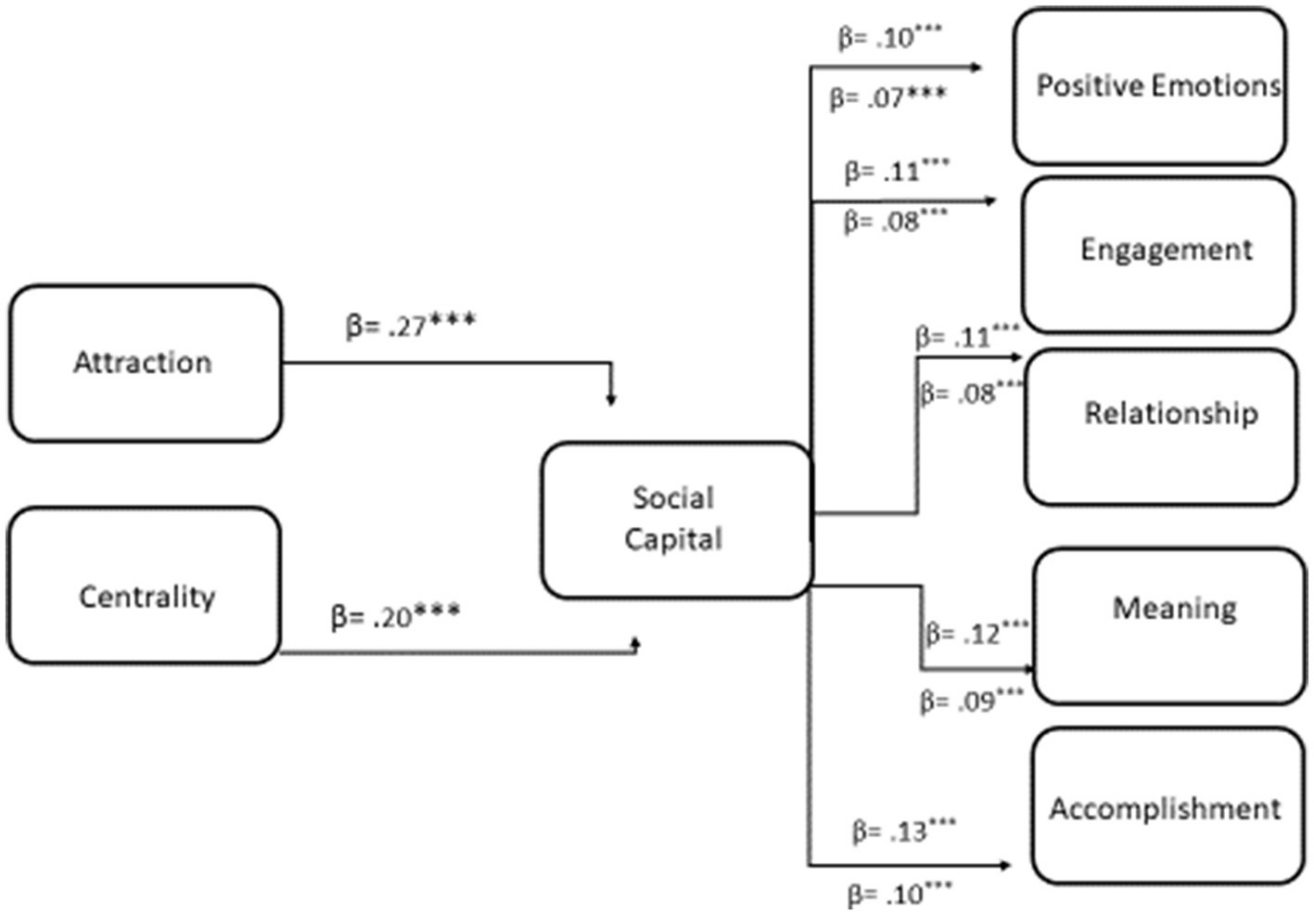
Mediation analysis of the association between attraction, centrality, and (i) positive emotions, (ii) engagement, (iii) relationship, (iv) meaning, and (v) accomplishment, by social capital. Note: ***p < 0.001.

A mediation analysis was conducted to examine whether Social Capital mediates the relationship between Attraction, Centrality, Self-Expression, and (i) Positive Emotions, (ii) Engagement, (iii) Relationship, (iv) Meaning, and (v) Accomplishment (Figure 2). The model demonstrated good fit, χ^2^ (9) = 32.17, *p* < 0.05 CFI = 0.99, TLI = 0.98, RMSEA = 0.07, 90% CI [0.04, 0.09], SRMR = 0.05. The results of the direct effects indicate that Attraction was a significant positive predictor of all outcomes: Positive Emotions (β = 0.26, *p* < 0.001), Engagement (β = 0.14, *p* < 0.001), Relationship (β = 0.18, *p* < 0.001), Meaning (β = 0.20, *p* < 0.001), and Accomplishment (β = 0.15, *p* < 0.001), suggesting that higher levels of at-traction are consistently associated with well being constructs. In contrast, Centrality did not significantly predict any of the outcomes (Positive Emotions: β = −0.02, *p* = 0.78; Engagement: β = 0.04, *p* = 0.36; Relationship: β = 0.05, *p* = 0.34; Meaning: β = 0.04, *p* = 0.52; Accomplishment: β = 0.05, *p* = 0.35), indicating no direct effect on these variables. Similarly, Self-Expression was not a significant predictor for any outcome (Positive Emotions: β = 0.01, *p* = 0.91; Engagement: β = 0.03, *p* = 0.59; Relationship: β = −0.09, *p* = 0.11; Meaning: β = −0.09, *p* = 0.10; Accomplishment: β = −0.07, *p* = 0.17). Attraction and Centrality presented also significant paths toward Social Capital, with β = 0.27, *p* < 0.001 and β = 0.20, *p* < 0.001, while Self-Expression did not show a statistically significant path with and β = 0.08, *p* = 0.15. Direct paths are represented in Table 3.

**Table 3:** Direct paths and corresponding standardized regression coefficients (β).

Predictor	Outcome	Estimate (β)	SE	*z*	*p*	95% CI
Attraction	Positive emotions	0.26	0.04	5.92	< 0.001	[0.18, 0.35]
Centrality	Positive emotions	−0.02	0.05	−0.28	0.78	[−0.12,0.09]
Self-expression	Positive emotions	0.01	0.05	0.11	0.91	[−0.10, 0.11]
Attraction	Engagement	0.14	0.04	3.57	< 0.001	[0.06, 0.22]
Centrality	Engagement	0.04	0.05	0.92	0.36	[−0.05, 0.14]
Self-expression	Engagement	0.03	0.05	0.53	0.59	[−0.07, 0.12]
Attraction	Relationship	0.18	0.05	3.93	< 0.001	[0.09, 0.27]
Centrality	Relationship	0.05	0.06	0.95	0.34	[−0.06, 0.16]
Self-expression	Relationship	−0.09	0.06	−1.61	0.11	[−0.20,0.02]
Attraction	Meaning	0.20	0.04	4.66	< 0.001	[0.12, 0.29]
Centrality	Meaning	0.04	0.05	0.65	0.52	[−0.07, 0.14]
Self-Expression	Meaning	−0.09	0.05	−1.63	0.10	[−0.19, 0.02]
Attraction	Accomplishment	0.15	0.04	3.75	< 0.001	[0.07, 0.23]
Centrality	Accomplishment	0.05	0.05	0.94	0.35	[−0.05, 0.15]
Self-expression	Accomplishment	−0.07	0.05	−1.38	0.17	[−0.17, 0.03]
Attraction	Social capital	0.27	0.04	6.33	< 0.001	[0.19, 0.36]
Centrality	Social capital	0.20	0.05	3.73	< 0.001	[0.10, 0.31]
Self-expression	Social capital	0.08	0.05	1.46	0.15	[−0.03, 0.19]

CI, Confidence Interval.

The results of the indirect effects through Social Capital indicate that Attraction significantly predicted higher levels of Positive Emotions (β = 0.10, *p* < 0.001), Engagement (β = 0.11, *p* < 0.001), Relationship (β = 0.11, *p* < 0.001), Meaning (β = 0.12, *p* < 0.001), and Accomplishment (β = 0.13, *p* < 0.001) via Social Capital, suggesting partial mediation since Attraction also had significant direct effects on these outcomes. Centrality also showed significant indirect effects on Positive Emotions (β = 0.07, *p* < 0.001), Engagement (β = 0.08, *p* < 0.001), Relationship (β = 0.08, *p* < 0.001), Meaning (β = 0.09, *p* < 0.001), and Accomplishment (β = 0.10, *p* < 0.001) through Social Capital, indicating total mediation where the direct paths were non-significant. In contrast, Self-Expression did not demonstrate significant indirect effects on any outcome (Positive Emotions: β = 0.03, *p* = 0.15; Engagement: β = 0.03, *p* = 0.15; Relationship: β = 0.03, *p* = 0.15; Meaning: β = 0.03, *p* =0.15; Accomplishment: β = 0.04, *p* = 0.15), suggesting no mediation via Social Capital (Table 4).

**Table 4:** Indirect paths and corresponding standardized regression coefficients (β).

Predictor	Mediator	Outcome	Estimate (β)	SE	*z*	*p*	95% CI
Attraction	Social capital	Positive emotions	0.10	0.19	5.11	< 0.001	[0.06, 0.14]
Centrality	Social capital	Positive emotions	0.07	0.21	3.42	< 0.001	[0.03, 0.11]
Self-expression	Social capital	Positive emotions	0.03	0.20	1.44	0.15	[−0.01, 0.07]
Attraction	Social capital	Engagement	0.11	0.20	5.49	< 0.001	[0.07, 0.15]
Centrality	Social capital	Engagement	0.08	0.23	3.53	< 0.001	[0.04, 0.13]
Self-expression	Social capital	Engagement	0.03	0.22	1.44	0.15	[−0.01, 0.08]
Attraction	Social capital	Relationship	0.11	0.21	5.30	< 0.001	[0.07, 0.15]
Centrality	Social capital	Relationship	0.08	0.24	3.48	< 0.001	[0.04, 0.13]
Self-expression	Social capital	Relationship	0.03	0.22	1.44	0.15	[−0.01, 0.08]
Attraction	Social capital	Meaning	0.12	0.21	5.41	< 0.001	[0.07, 0.16]
Centrality	Social capital	Meaning	0.09	0.24	3.51	< 0.001	[0.04, 0.13]
Self-expression	Social capital	Meaning	0.03	0.23	1.44	0.15	[−0.01, 0.08]
Attraction	Social capital	Accomplishment	0.13	0.23	5.64	< 0.001	[0.08, 0.17]
Centrality	Social capital	Accomplishment	0.10	0.27	3.57	< 0.001	[0.04, 0.15]
Self-expression	Social capital	Accomplishment	0.04	0.26	1.45	0.148	[−0.01, 0.09]

CI, Confidence Interval.

The total effect of Attraction on all outcomes was positive and statistically significant. Specifically, Attraction positively predicted Positive Emotions (β = 0.36, *p* < 0.001), Engagement (β = 0.25, *p* < 0.001), Relationship (β = 0.29, *p* < 0.001), Meaning (β = 0.32, *p* < 0.001), and Accomplishment (β = 0.28, *p* < 0.001). Centrality showed smaller but significant total effects on Engagement (β = 0.13, *p* = 0.017), Relationship (β = 0.14, *p* = 0.022), Meaning (β = 0.12, *p* = 0.037), and Accomplishment (β = 0.14, *p* = 0.011), while its effect on Positive Emotions was not significant (β = 0.06, *p* = 0.319). Self-Expression did not have significant total effects on any outcome, including Positive Emotions (β = 0.03, *p* = 0.55), Engagement (β = 0.06, *p* = 0.276), Relationship (β = −0.06, *p* = 0.341), Meaning (β = −0.05, *p* = 0.357), and Accomplishment (β = −0.03, *p* = 0.575). These results suggest that Attraction consistently has the strongest overall influence on well being outcomes, Centrality exerts moderate effects, and Self-Expression shows negligible total effects. Figure 2 depicts the significant indirect effects.

## 5 Discussion

This study aims to test whether involvement with an extreme group conditioning program influences the development of subjective well being, considering social capital as a mediator variable.

### 5.1 The relationship between involvement and social capital

As has been reported, participants in group extreme conditioning program training are particularly committed and involved with their training programs (Shuda and Feito, 2017). The results of the present study indicated that such training is perceived as pleasurable and enjoyable, which are the indicators of the first dimension of involvement named Attraction. The results also showed that participants perceive such training as an important part of their life, having scored high on the Centrality dimension of involvement. Finally, extreme conditioning program training is used as a means of expressing their personal identity, which is built within the social connectivity within the training groups. This is expressed by the Self-expression dimension of involvement. In terms of the relationship between involvement and social capital, the results indicated that the attraction and centrality dimensions had statistically significant and moderate to strong associations with social capital. This is in line with previous research in which it was reported that extreme conditioning program training has a strong social positioning and community-driven ethos (Gao et al., 2025). As previously noted, Putnam (1995) conceptualized social capital based on three core elements: networks, trust, and reciprocity. Networks in group extreme conditioning program training settings are developed as participants, who share common interests (e.g., strength and conditioning training) and values (e.g., healthy lifestyle), form onsite training groups, which can be perceived as a “second family” (Whiteman-Sandland et al., 2016), all of which address SDG 3 and SDG 16. These training groups can be extended into networks, including coaches and affiliated clubs, but also online groups and blogs. Reciprocity is developed when the group-based training format promotes cooperation and mutual help in training to achieve personal goals (Prayitno et al., 2024). Reciprocity can be linked with the notion of solidarity as well, through collective effort and shared trials during exercise (Ari et al., 2024). Members of extreme conditioning program training groups “give and receive”, as they help each other in achieving the escalated training goals. This creates an exchange system that helps the development of trust among the members of the groups, as defined by SDG16. Participants develop common norms as they belong to the “extreme conditioning program training family,” and these norms facilitate cooperation at a community level (Ari et al., 2024). Social support was reported as the main motivator in the studies of Whiteman-Sandland et al. (2016) and Ntovoli et al. (2025). The absence of a significant relationship between self-expression and social capital is a noteworthy finding that warrants further investigation. Self-expression typically reflects the extent to which individuals use an activity to communicate their identity, values, or lifestyle to others. However, this identity-related dimension of involvement may not necessarily translate into the formation of trust, reciprocity, or network cohesion—core components of social capital. It is possible that participants express their identity through the activity itself rather than through social interaction, or that identity-driven motives operate independently of the social structures within group extreme conditioning programs. Future research should explore these dynamics more closely, examining whether self-expression contributes to different forms of social capital, operates through alternative mechanisms, or is influenced by contextual factors such as group norms, cohesion, or the competitive nature of the training environment.

### 5.2 The relationship between social capital and well being

As previously discussed, social capital has been associated with increased physical, social, and psychological health for individuals, but also community building, contributing finally to healthy societies (Forsell et al., 2020). The results of our study supported and further extended these findings as they provided evidence that social capital is strongly associated with all the dimensions of PERMA. Members of group extreme conditioning program training networks develop and accept common norms, build trust and reciprocity, because of exercise engagement, and are more likely to perceive higher levels of subjective well being, addressing SDG3. Extreme conditioning activities are delivered in settings that help individuals acquire benefits and assets in relation to networking and trust, which are important elements of social capital (Zhou et al., 2021).

As previously noted, scalability is a distinctive feature of extreme conditioning program training that fosters autonomy and progressive mastery (Davies et al., 2016). This element influences the development of the Accomplishment and Positive Emotions dimension of PERMA. Davies et al. (2016) reported that 65% of participants enhanced self-efficacy after 6 months of training. The group nature of training develops community ethos and trust among members, which can influence the development of the Relationship and the Meaning dimensions of PERMA. These are further supported by several charity events, which are common practices among exercise clubs and teams.

### 5.3 The relationship between involvement and well being

One of the objectives of the study was to test how important social capital is in terms of the development of subjective well being, by testing its mediating role. First, the results showed that social capital fully mediated the relationship between centrality and subjective well being. Centrality had only an indirect relationship with the dimensions of well being through social capital, which indicates the key role that social capital plays in the development of perceived well being. Individuals highly involved in extreme conditioning program training are more likely to report higher levels of well being, only when they develop trust and reciprocity through group training and networking. However, the results also showed that social support partially mediated the relationship between attraction and well being, showing both direct and indirect relationships. Attraction is therefore a strong dimension of involvement, as has also been reported in previous research in other sport settings (Alexandris, 2013).

## 6 Conclusion

This study, for the first time, provided empirical evidence for the important role of social capital in achieving high levels of individual well being and collective societal well being in the context of extreme conditioning program training, addressing SDG3 and SDG16. It can therefore be argued that the wide expansion of such exercise programs and the development of a committed participant base is not only due to the physical benefits, as previous research has shown (Claudino et al., 2018), but also to the positive social and psychological outcomes. They also provide support for the marketing strategy to position such an exercise as a community and social activity that fosters trust, reciprocity, and promotes social cohesion among participants, promoting certain SDGs.

## 7 Study limitations and future research

As previously noted, the study followed a cross-sectional design. This means that causal relationships revealed in the structural model should be interpreted with caution. A longitudinal approach can give more trust to examine causal relationships and test the sustainability of the positive outcomes. A second issue that should be noted relates to the position of social outcome within the structural model. We hypothesized that social capital is an outcome of involvement that predicts subjective well being. As previously noted, social capital can be both an outcome and an antecedent of sport participation (Kim et al., 2021). Future studies could test social capital as an antecedent of sport involvement and even as an antecedent of well being. A final note should be made about the cultural element of the study. A Greek sample of an extreme conditioning program training was used. Testing the theoretical model in a sample of individuals from different cultures would give us more trust to make more reliable generalizations.

## Data Availability

The datasets presented in this article are not readily available because We stated in the consent form and the ethics committee that the data is confidential. Requests to access the datasets should be directed to Apostolia Ntovoli/antovoli@phed.auth.gr.
